# Bell’s palsy or an aggressive infiltrating basaloid carcinoma post-mRNA vaccination for COVID-19? A case report and review of the literature

**DOI:** 10.17179/excli2023-6145

**Published:** 2023-09-15

**Authors:** Anthony M. Kyriakopoulos, Greg Nigh, Peter A. McCullough, Maria D. Olivier, Stephanie Seneff

**Affiliations:** 1 Director and Head of Research and Development, Nasco AD Biotechnology Laboratory, Department of Research and Development, Sachtouri 11, 18536, Piraeus, Greece; 2 Naturopathic Oncologist, Immersion Health, Portland, OR 97214, USA; 3 Chief Medical Advisor, Truth for Health Foundation, Tucson, AZ, USA; 4 Director and medical practitioner, Dr. Maré Olivier, Inc., Kuils River, South Africa; 5 Senior Research Scientist, Computer Science and Artificial Intelligence Laboratory, Massachusetts Institute of Technology, Cambridge, MA, USA

**Keywords:** mRNA vaccines, basal cell carcinoma, Bell's palsy, exosomes, metastatic malignancy, autoimmunity

## Abstract

We report on an aggressive, infiltrating, metastatic, and ultimately lethal basaloid type of carcinoma arising shortly after an mRNA vaccination for COVID-19. The wife of the patient, since deceased, gave the consent for publishing the case. The malignancy was of cutaneous origin and the case showed symptoms consistent with Bell's palsy and trigeminal neuralgia beginning four days post-vaccination (right side head temporal pain). The temporal pain was suggestive for inflammation and impairment of T cell immune activation. Magnetic Resonance Imaging (MRI) showed a vascular loop on the left lateral aspect of the 5^th^ cranial root exit of cerebellopontine angle constituting presumably a normal variant and was considered as an unrelated factor to the right-sided palsy and pain symptoms that corresponded to cranial nerves V (trigeminal nerve) and VII (facial nerve). In this study we describe all aspects of this case and discuss possible causal links between the rapid emergence of this metastatic cancer and mRNA vaccination. We place this within the context of multiple immune impairments potentially related to the mRNA injections that would be expected to potentiate more aggressive presentation and progression of cancer. The type of malignancy we describe suggests a population risk for occurrence of a large variety of relatively common basaloid phenotype cancer cells, which may have the potential for metastatic disease. This can be avoidable with early diagnosis and adequate treatment. Since facial paralysis/pain is one of the more common adverse neurological events following mRNA injection, careful inspection of cutaneous/soft tissue should be conducted to rule out malignancy. An extensive literature review is carried out, in order to elucidate the toxicity of mRNA vaccination that may have led to the death of this patient. Preventive and precise routine clinical investigations can potentially avoid future mortalities.

See also Figure 1(Fig. 1).

## Introduction

Bell's palsy is a relatively common condition affecting up to 40,000 people just in the United States every year. It typically manifests as a sudden onset unilateral facial paralysis. Most people recover fully after several months, but the damage can become permanent. It is due to inflammation in the facial nerve, leading to nerve injury. The cause of inflammation is multifactorial, and herein lies the danger of a missed diagnosis of an underlying condition that could be life threatening and would be treatable if diagnosed sufficiently early (Bacorn et al., 2020[13]; Zhang et al., 2020[93]; Nguyen et al., 2021[61]). 

Bell's palsy is considered to be an idiopathic disease, and the diagnosis must be done carefully so as to exclude potential etiologies of facial paralysis such as trauma, neoplasm, or viral infection (Zhang et al., 2020[93]). Approximately 80 % of cases of facial paralysis are idiopathic, and the expectation is of full recovery. But 20 % can be due to an underlying condition, most ominously, an occult facial nerve neoplasm. In such cases, the cancer can be overlooked, and this can lead to delayed treatment with serious consequences (Chung et al., 2022[24]). 

Bell's palsy is frequently associated with a viral infection, most commonly Herpes simplex, Epstein-Barr virus, Varicella-zoster, and Cytomegalovirus (Greco et al., 2012[33]). Several case studies have been reported in the literature linking SARS-CoV-2 infection with Bell's palsy (Alrajhi et al., 2022[7]; Guan et al., 2020[35]; Nath, 2020[60]; Mao et al., 2020[53]; Cabrera Muras et al., 2021[20]), and the mechanisms may be similar to those involved with other viruses. A common element is likely molecular mimicry leading to an autoimmune attack on the facial nerve. 

The mRNA SARS-CoV-2 vaccines have also been linked to Bell's palsy (Khurshid et al., 2022[44]). A review paper identified 17 studies linking COVID-19 vaccines to Bell's palsy, with the Pfizer-BioNTech and Moderna mRNA vaccines being most commonly associated (Shahsavarinia et al., 2022[74]). One likely hypothesis for a causal relationship is neural ischemia due to blockage of blood flow because of hypercoagulability induced by the spike protein (Alrajhi et al., 2022[7]). It has been shown experimentally that the S1 segment of the spike protein induces the formation of fibrin micro-clots that are highly resistant to fibrinolysis (Grobbelaar et al., 2021[34]). Another possibility is an attack on the facial nerve due to molecular mimicry (Alrajhi et al., 2022[7]). Several human proteins that are involved in maintenance of the myelin sheath have been found to share pentamers with the spike protein that could lead to autoimmune attack on the myelin sheath via molecular mimicry (Cuspoca et al., 2022[26]). This is therefore similar to the underlying etiology for facial paralysis linked to other viral infections. 

In this paper, we describe the case of a man who was diagnosed with Bell's palsy shortly after receiving his first and only SARS-CoV-2 mRNA vaccine. Because Bell's palsy is a known side effect of the vaccine, it was not determined until much later that he suffered from infiltrating metastatic cancer that had spread to the facial nerve, a likely causal factor in his paralysis symptoms. A previous case study involving a 78-year-old man parallels our case in many respects (Viken et al., 2011[84]). This man had been treated for a skin cancer in the right buccal region of his face, diagnosed initially as basal cell carcinoma, later suggested to be squamous cell carcinoma. After a dermatologist had declared his skin cancer to be cured, he developed pain in the region supplied by the maxillary branch of the trigeminal nerve. Despite treatment, the pain persisted, and he developed third and sixth cranial nerve palsies, and complete loss of sensory function of the right trigeminal nerve, along with temporal muscle atrophy and ninth cranial nerve palsy. Although the evidence provided by MRI was difficult to read, his condition was ultimately diagnosed as cranial nerve spread of his original skin cancer. He passed away shortly thereafter from pneumonia. 

Cancers of the head and neck have a propensity to invade nerves, and to use nerves as a route for tumor spread. Spread is mostly to the trigeminal nerve and the facial nerve, and the most common types of cancer that spread in this way are squamous cell carcinoma, adenoid cystic carcinoma, and salivary duct carcinoma (Amit et al., 2016[10]). 

In the following sections we describe in detail our patient's disease progression, and we develop several hypotheses to support the argument that the vaccine may have accelerated his decline. We propose that the vaccine can cause suppression of the immune system, which leads to accelerated cancer progression. The elevated D-dimers in our patient's case are prognostic for CD8+ T cell downregulation and poor outcome in cancer (Li et al., 2022[49]; Guan et al., 2022[36]). We provide evidence from the Food and Drug Administration's Vaccine Adverse Event Reporting System (VAERS) to show that several symptoms that were experienced by our patient are over-represented in adverse events associated with COVID-19 vaccines compared to all other vaccines. 

## Case Report

A 56-year-old white male with no significant previous history of medical conditions, but with a family history of malignancy, (mother had lung cancer due to smoking), presented with a massive and aggressively infiltrating basaloid-featured cancer in the right side of his face that rapidly evolved, ultimately ending in the patient's death. Symptoms began four days after his first mRNA vaccination (Pfizer), when he presented with severe right-sided temporal pain spreading to the temporomandibular (TM) joint. As the pain continued unabated and even worsened over the next two months despite the use of non-steroidal anti-inflammatory drugs, the patient underwent a brain MRI examination. The MRI did not reveal any pathological features. A vascular loop was identified at the left side of cranial nerve V (trigeminal nerve) root and was considered as a normal variant. Given its left-sided location it was not considered a contributing factor to the patient's pain. The pain progression was accompanied with ptosis of the right eyelid. This was suggestive at the time as an initial symptom of Bell's palsy and considered a common side effect induced by the mRNA vaccines for COVID-19 by a consultant neurologist. 

**Figure 2:**
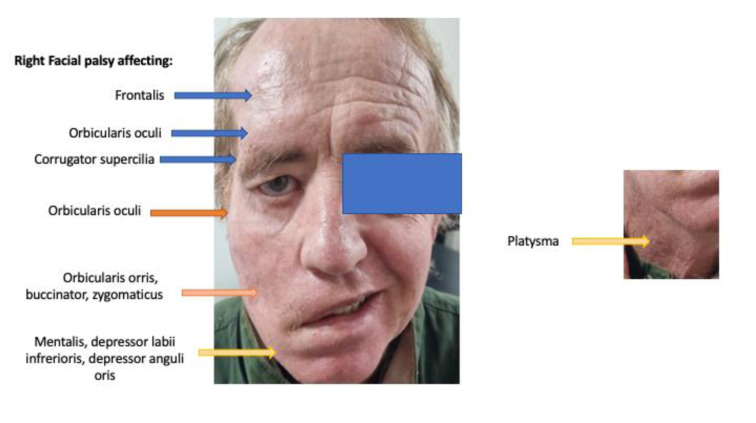
Patient showed unilateral right facial palsy seemingly affecting all branches of cranial nerve VII. This was his condition 5 months post the mRNA vaccination.

Two months later, and as the condition worsened, the patient was diagnosed as suffering from right-sided trigeminal neuralgia and cranial nerve VII palsy. The right-hand side of the face showed a semi-unilateral palsy presenting as a lower motor neuron malfunction of all branches of the facial nerve (temporal, zygomatic, buccal and mandibular branches; Figure 2(Fig. 2)). Subsequent developments in this case, as we describe below, suggest this represented a lesion of the facial nerve at the cranial exit root. The patient began receiving intravenous vitamin C, plus oral vitamin D, quercetin, melatonin, zeolite and, after a week, reported dramatic improvement in pain and weakness symptoms.

**Figure 3:**
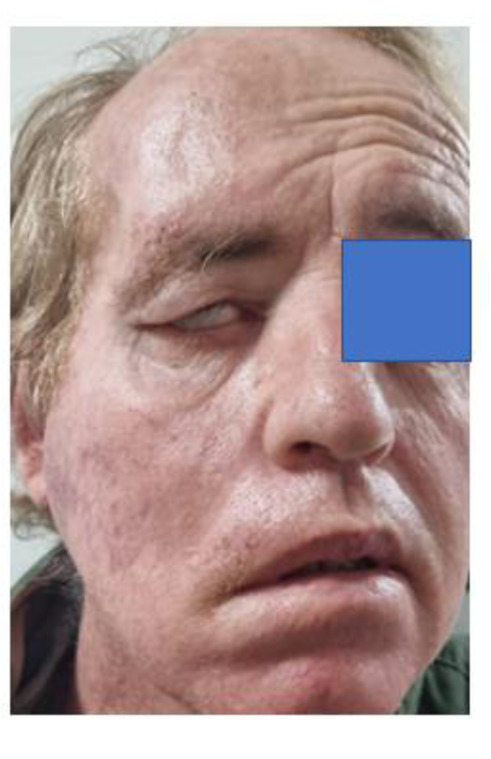
Progressive inability to open the right eye and right side of mouth. This was his condition 7 months post the mRNA vaccination.

**Figure 4:**
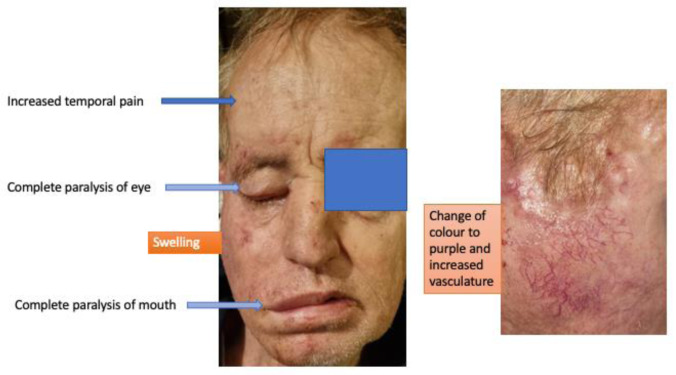
Total paralysis of right face with increased swelling, change of skin color, with increased vascularity. This was his condition 11 months post the mRNA vaccination.

Another two months later the patient was showing progressive wasting and loss of weight (12 kg) with progressing facial palsy symptoms (Figure 3(Fig. 3)). Four months after that - now 8 months post-mRNA injection - the patient showed increased temporal pain, deafness in the right ear, progressive wasting, inability to open the right eye, inability to open the right side of his mouth, vertigo, and limb weakness (Figure 4(Fig. 4)). A total paralysis of the right face was recorded with increased pain, swelling of the face on the right side, change of skin color to purple, and increased vascularity. Otoscopy of the right ear was not feasible due to an extensive protruding mass blocking the external auditory canal. Sensation was by this time absent in both the external ear and the canal on that side. The patient's only hematological examination due to his lack of financial resources was on D-dimers which was performed at this time. The D-dimer value was exceedingly high, 1523 ng/ml, with the normal range being < 500 ng/ml. A high D-dimer level is associated with poor outcome in cancer patients (Ay et al., 2012[12]). 

**Figure 5:**
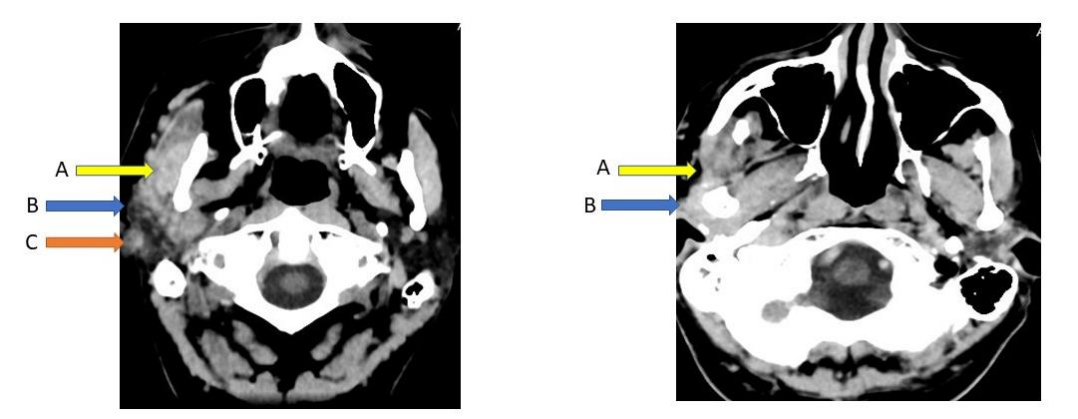
Diffuse mass-like nodularity of right parotid gland superficial and deep portion with swelling, loss of normal plane of separation between the parotid gland and masseter muscle (A). Overlying skin thickening extending up to the inferior aspect of the right ear (B). Small quantity of non-specific soft tissue in right middle ear cavity (C).

The patient's family doctor recommended a computerized tomography (CT) brain scan and biopsy. The CT scan revealed a diffuse tumor of the right parotid gland (both superficially and in the deep portion), with swelling. There was loss of the normal separative space between the parotid gland and masseter muscle with marked swelling of the muscle and overlying skin thickening up to the inferior aspects of the right ear (Figure 5(Fig. 5)). However, the CT scans were not conclusive of any tumor within the brain parenchyma or impinging on it. Nor could any other pathological findings be found in proximity to cranial nerves V and VII pontine nuclei that might explain facial palsy, sensations (loss of feeling in the right ear), and other symptoms. Biopsy revealed multiple infiltrations of basaloid type cell islands within the zygomatic, temporomandibular joint, optic nerve, fifth cranial nerve, and greater auricular nerve (data not shown but histology report is provided in supplementary information). The tumor cells had no connection to the basal cells or other layers of the epidermis. The infiltrations of cancer cells were extensive, and therefore the margins throughout the periphery of the tumor were not clear. Hence, surgical excision of the tumor was not considered a viable option. The patient's condition was not treated by any other means apart from morphine administration, and ultimately the patient passed away 4 months after the biopsy. 

### Biopsy

The biopsy specimens delivered for histopathology examination were topographically unlabeled and consisted of multiple tissue fragments (3 x 1 x 1 cm). The dermis consisted of core fragments presenting considerable actinic elastosis. Infiltrations were composed of basaloid type cells with no connection to the overlying epidermis. Small areas of low refractility indicated separation of tumor islands from adjacent tissue. No obvious squamous type of cell involvement was evident, nor was there any evidence of parotid parenchyma. Supplementary note of histological evaluation (provided in supplementary information) stated that there were infiltrations into the zygomatic, temporomandibular joint, optic nerve, fifth cranial nerve, greater auricular nerve (perineural invasion or perineural spread), as well as widening of the foramen ovale, and mandibular lesions. Due to the widespread marginal infiltrations of the tumor, the condition was considered to be inoperable.

### Differential diagnosis of tumor

Basal cell carcinomas (BCCs) rarely progress to metastatic disease. They are more frequently diagnosed in males (Mehta et al., 2012[57]). Moreover, although BCC metastases are rare, an early diagnosis is essential to prevent their increased morbidity and mortality (Piva de Freitas et al., 2017[66]). In the present case, although the patient's skin suffered from solar elastosis, the patient's tissue biopsy revealed (data not shown) that the tumor did not involve attachment to layers of the epidermis. This strongly suggests it was of cutaneous origin. In order to investigate the aggressiveness of the tumor and capabilities of the vigorous infiltration observed in this type of basaloid-type cancer, an analysis of malignant cell tissue markers in biopsy can be revealing. The tumor cells were negative for epithelial membrane antigen (EMA) and, according to Villada et al. (2018[85]) this diminishes the possibility that this was a basaloid squamous cell carcinoma (BSCC) and enhances the possibility of a basal cell cancer. 

However, there are many clinical variants of BCC (Dourmishev et al., 2013[28]) and other cancers that can present with a basaloid phenotype, such as poorly differentiated squamous cancer cells (Winters et al., 2008[89]). Basaloid squamous cancer cells have a lower expression of EMA (15 % are negative) when they form a neoplasm of lower differentiation (Tatemoto et al., 1987[82]). Moreover, the percentage of EMA lack of expression and hence negativity during the antibody staining is higher when the tumor originates from nonkeratinized lesions, as was the case here. It should be noted that ulcerating lesions in the preauricular area were not investigated during the biopsy. 

Furthermore, the basaloid type tumor cells in this patient's biopsy report were strongly positive under Ber-Ep4 histological staining, indicating their strong expression of epithelial glycoprotein cell adhesion molecule (EpCAM). Ber-Ep4 stain is used extensively to differentiate basal cell carcinomas that strongly express the EpCAM from other types of basaloid cancers (Sunjaya et al., 2017[81]). A poorly differentiated squamous cell carcinoma can sporadically express EpCAM and be positive for Ber-Ep4 antibody stain (Webb et al., 2015[88]), and at the same time not express EMA (Tatemoto et al., 1987[82]). Therefore, the presence of a basaloid squamous cell carcinoma (BSCC) of low differentiation cannot be ruled out with certainty in this case. 

Also, the possibility of this malignancy being a metatypical (squamoid) basal cell carcinoma (MBCC) cannot be excluded, as this type of cancer is also strongly positive in Ber-Ep4 stain (Webb et al., 2015[88]). The application of a MOC-31 monoclonal antibody to identify the expression of a 40kd glycoprotein would have helped eliminate the possibility that this was a squamoid basaloid type of cancer cells (Ruitenbeek et al., 1994[69]; Webb et al., 2015[88]). Beyond that, the strong positive signal of Ber-Ep4 in the absence of EMA expression, although characteristic of BCC, is also encountered in trichoepithelioma (TE). BCCs can simulate the pattern of TEs. Therefore, the use of CD10 staining, in the absence of EMA expression, could have differentiated further the BCC as an underlying TE-like BCC (Carr et al., 2007[21]). In most respects, the description of tumor islands in this case followed a peripheral palisading motif and stromal clefting, which is strongly suggestive that this was purely a basaloid cell carcinoma rather than a TE in a less organized variant version (Dourmishev et al., 2013[28]).

Stromal clefting is in fact an artefact observed during the microscopical examination and corresponds to peritumoral mucin deposition (Ulrich et al., 2011[83]) seen in BCCs. However, increased stromal mucin can also be observed in sweat gland carcinomas with metastasis to the parotid gland (Sarangi et al., 2022[70]). Sweat gland carcinomas constitute a rare low-grade adnexal neoplasm of cutaneous origin entity that, when it presents basaloid formations, can be mistaken for BCC (Obaidat et al., 2007[62]). In most cases of adnexal carcinomas, however, the EMA immunocytochemistry stain is positive (Macagno et al., 2022[52]), but the invasive phenotype in these cases (Sarangi et al., 2022[70]) mimics the invasiveness of our case's cancer, especially under the CT scan examination. 

The invasion in the preauricular region, masseter, parotid gland, and mandibular ramus of an endocrine mucin-producing sweat gland carcinoma (EMPSCG) case mimics overwhelmingly the current case we report here (Sarangi et al., 2022[70]). EMPSCG develops primarily in the eyelids of elderly patients and causes devastating metastatic disease. Due to lack of further biopsy examinations, we can only say that the phenotype (metastatic potential) of basaloid featured cancer in our case showed a close resemblance with EMPSCG (Sarangi et al., 2022[70]). For an infiltrating BCC that has no connection to epidermal layers to show so much close resemblance to EMPSCG is extremely rare (Dourmishev et al., 2013[28]). 

However, further neuroendocrine markers would have helped to preclude the presence of an adnexal type of cancer in this report (Obaidat et al., 2007[62]). Finally, but not least, the p63 positivity of the basaloid cells in this case is consistently expressed in basal, suprabasal and adnexal basal tumor cells, strongly suggesting a common basaloid progenitor cell lineage responsible for developing BCC (Bircan et al., 2006[16]). The basaloid squamous cell carcinomas show an inconsistency in expressing p63 (diffuse p63 positivity), signifying a p63 dysregulation in the stem progenitor cells, producing a poorly differentiated type of this tumor. Additionally, the cytokeratin 5 (CK5), which is found positive in our case's basaloid type of cells, is diffusely positive in BCCs, whereas the metastatic squamous cell carcinomas of epidermal origin strongly express CK5 (Johansson, 2004[41]).

In summary, this was an infiltrating type of BCC originating from a cutaneous site of skin that mimicked the clinical course of an aggressive EMPSCG recently described (Sarangi et al., 2022[70]). In many respects, the presence of an adnexal type of basaloid-type neoplasm cannot be ruled out in this case due to their common resemblances in infiltration, aggression, and invasive characteristics.

### Differential diagnosis of Bell's palsy, pain, and trigeminal neuralgia 

**Figure 6:**
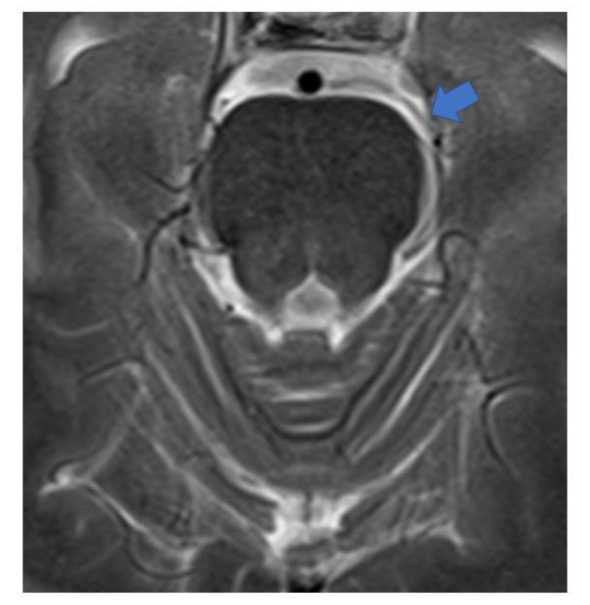
High resolution axial MRI image. The vascular loop runs alongside the left lateral aspects of cranial nerve V (trigeminal) root exit.

**Figure 7:**
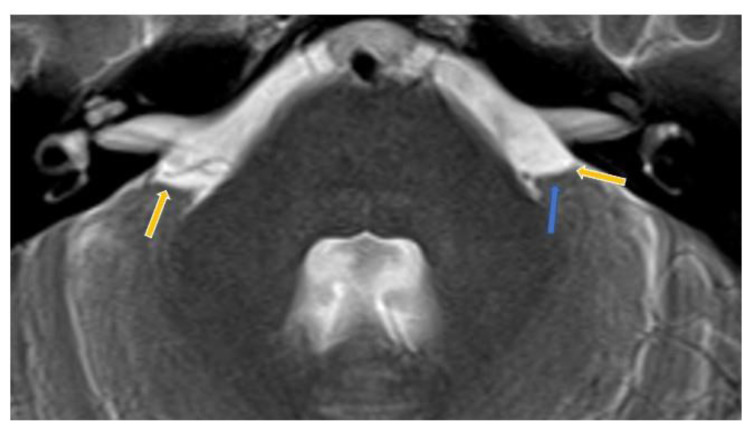
The left trigeminal V nerve cannot be well visualised as the vascular loop (blue arrow) hides the lateral structures of the left cerebellopontine angle as compared to the right sided trigeminal V nerve (yellow arrows).

The patient developed right temporal pain four days after his first vaccination with mRNA (Pfizer) against COVID-19. During the next two months, the right temporal pain on the upper face became severe, and the symptoms were compatible with trigeminal neuralgia in the ophthalmic and maxillary regions (Anwar et al., 2022[11]). The MRI conducted at that time showed a vascular loop approximating to the lateral side of the cranial nerve V exit root (Figures 6(Fig. 6) and 7(Fig. 7)). Otherwise, the brain parenchyma was clear of the presence of any kind of tumor growth; see Figure 7(Fig. 7). Vascular loops are indicative for compression of cranial nerves at the site of root exits, and they are associated with trigeminal neuralgia (Baldauf et al., 2019[14]). Finally, the patient progressed to complete sensorineural loss of hearing well before the auricle canal was blocked by the growing tumor mass. 

In this regard, vertigo, tinnitus and hearing loss from which our patient suffered are found to be connected with vascular loops (Chadha and Weiner, 2008[22]). Furthermore, the surgical de-compression of cochleovestibular vascular compression can result in complete abolishment of tinnitus (Brookes, 1996[19]). However, later studies argued that the detection of vascular loops by MRI scanning is not an etiological factor for tinnitus (Gultekin et al., 2008[37]). The symptom of tinnitus that our patient suffered from developed six to eight months after the mRNA vaccination. The tinnitus accompanied the loss of hearing. Similar studies indicate that these symptoms do associate with vascular loops in the cerebellopontine angle and can be due to underlying developing tumors. Additionally, the two symptoms together, tinnitus and sensorineural hearing loss (I can't feel my ear), as described by the patient during clinical examination, liaise with vascular loops (Moosa et al., 2015[59]; Hofmann et al., 2013[39]; Ramly et al. 2014[68]). 

According to a study by Anwar et al. (2022[11]), the majority of neurovascular contacts (NVC) cause trigeminal neuralgia on the ipsilateral side. However, in the same study it was also shown that 34 % of the patients suffering from trigeminal neuralgia had NVC at the contralateral trigeminal nerve. Anatomically, the main sensory nucleus (MSN) lies in the pons adjacent to the entering root fibres, and there is a somatotopic organisation to cause contralateral pain disorders (Walker et al., 1990[87]). A high percentage of ascending fibres from the MSN travel with the contralateral median lemniscus (ML), ending in the ventral posteromedial (VPM) nucleus of the thalamus. 

The patient also developed limb weakness and loss of discriminative touch around the ear; hence, the case of ML damage upstream of the exit of cranial nerve V cannot be excluded, as ML is implicated in contralateral symptoms from vascular defects (Siddik and Gupta, 2022[77]). Therefore, the possibility that our patient was suffering from trigeminal neuralgia due to vascular compression of the left cranial nerve V root and that the trigeminal nerve was harmed upstream from the site of symptomology cannot be excluded, since the patient's initial pain complaints originated in the sensory root of the trigeminal nerve. 

The case of ischemic stroke at first signs of facial palsy was dismissed. The early facial weakness of our patient reflected the nerve branches at both the upper and lower parts of the face (see Figures 2(Fig. 2), 3(Fig. 3) and 4(Fig. 4)). An ischemic stroke resulting from lesions of a damaged motor cortex would have shown a contralateral facial weakness of the lower face only. In this regard, the nerve branches that control the muscles of the upper face on both sides travel from the cortex, and half of them cross over in the brainstem to the contralateral facial nerve, whereas the other half continue to exist on the same side and signal to the ipsilateral facial nerve. 

During an ischemic stroke there will be preservation of neural signaling to the muscles of the upper face on both sides (although weakened on one side) due to a double reaction on the upper face, and the patient would have had symmetrical forehead wrinkling and would be able to close the eye tightly. Our patient had a right forehead diminution of wrinkling (see Figure 3(Fig. 3)), and complete paralysis of muscles controlled by ophthalmic facial nerve branches (see Figure 4(Fig. 4)) (Marfeo, 2010[54]). The facial palsy symptoms were regarded as peripheral lesions affecting the facial nerve as it exited from the brainstem. The facial palsy of our patient reflected all (temporal, zygomatic, buccal mandibular and platysma) branches of the facial nerve (see Figure 4(Fig. 4)) and followed all the criteria to be diagnosed as Bell's Palsy (Fahimi et al., 2014[30]).

**Figure 8:**
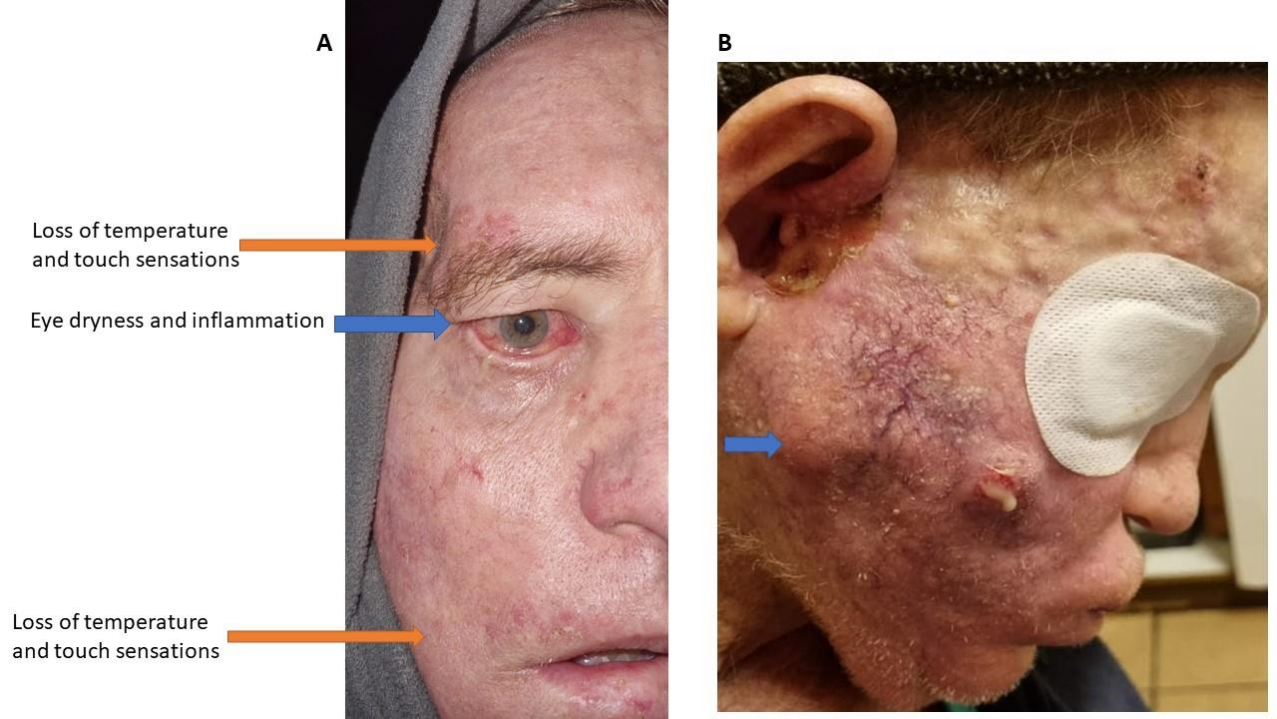
Eye dryness and irritation. Loss of temperature and touch sensations in ophthalmic and mouth regions. Condition 5 months post the mRNA vaccination (A). Skin necrosis and purulent exudates probably due to secondary bacterial infection. Remarkable change of skin color, edema and increased vascularity. Preauricular cavitation starting to form with inflammatory exudates. Growing mass on zygomatic region (blue arrow). Condition 13 months post the mRNA vaccination (B).

**Figure 9:**
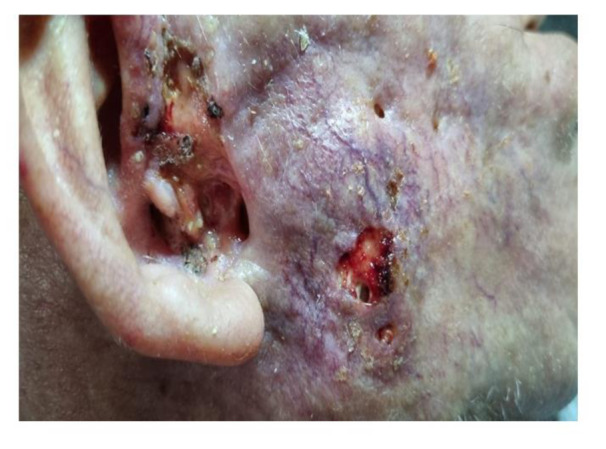
Extreme cavitation in the preauricular region, extreme necrosis of the skin and extensive infiltrations of vasculature. Condition 15 months post the mRNA vaccination.

In parallel and before the eye muscles progressed to a complete paralysis, there was a remarkable eye dryness and irritation. This coexisted with upper eyelid skin inflammation (Figure 8A(Fig. 8)). In the same region of upper lid inflammation, there was increased cranial nerve pain both in the past and during the clinical examination, according to the patient. Finally, during the initial stages of Bell's palsy, the patient's skin showed increased vascularity in the right facial region where the facial nerve branches to its temporal, zygomatic, buccal and mandibular sub-segments (see Figure 4(Fig. 4)). As the case progressed, this area got increasingly swollen, produced purulent exudates (suggesting a secondary bacterial infection) and skin necrosis (Figure 8B(Fig. 8)), ultimately progressing to an extreme cavitation in the preauricular area with extensive necrosis of the skin (Figure 9(Fig. 9)). In conclusion, the prodromal trigeminal neuralgia could have co-existed with Bell's palsy that was underlying the tumor's development. In this regard, symptoms of Bell's palsy have long been shown to co-exist with progressive tumors, as in the case of invasive basal cell carcinoma (May and Lucente, 1972[56]).

## Data Analysis and Literature Review

### Analyses from the VAERS database 

The United States Food and Drug Administration (FDA) has maintained a publicly available database of vaccine reactions for all vaccines administered since 1990 (https://wonder.cdc.gov/vaers.html), and it has become one of the most important resources for discovering potential adverse reactions in the post-marketing phase (Shimabukuro et al., 2015[76]). In a previous publication, we provided several tables tabulating vaccine adverse events for COVID-19 vaccines compared to all other vaccines, focusing on the year 2021 (Seneff et al., 2022[72]). Since 2021 was the first year when COVID vaccines became available, as well as the year in which a massive campaign was undertaken to get much of the population immunized against COVID-19, 2021 is a good year to choose for analysis of vaccine injuries linked to COVID-19 vaccines. In that publication, we also compared the rate of vaccine injuries per vaccine administered in 2021 for both the COVID-19 vaccines and the flu vaccines. We estimated that there were 27 times as many reports per vaccine associated with the COVID-19 vaccines compared to those associated with the flu vaccine. We also estimated the percent of adverse reactions among all vaccines that would be expected to be attributed to COVID-19 vaccines, given the available knowledge that there were overall about three times as many vaccines administered for COVID-19 compared to flu shots that year, and assuming that the number of reactions to COVID-19 vaccines was comparable to the number of reactions to flu shots. This number came out to 32.6 % of all events, as contrasted with the reality that 93 % of all reactions were attributed to COVID-19 vaccines.

Table 1(Tab. 1) shows the number of reactions reported for COVID-19 vaccines, compared to all vaccines in 2021, for a number of different reactions that are in some way related to our case study. For some of the entries, we combined several adverse events to tally up the total number. For instance, there were a total of 45 different unique reactions containing either the word “metastasis” or the word “metastatic,” and these were all combined to give the total tally of 189 cases of associations of COVID-19 vaccine with metastasis. There were only 3 cases linking any other vaccines to metastasis in the year 2021. For “D-dimer,” we included reactions labeled as “D-dimer” and “D-dimer elevated.” As the table shows, for “carcinoma,” “D-dimer,” “metastasis,” “neck mass,” “neoplasm,” and “tinnitus,” over 98 % of the cases were associated with COVID-19 vaccines. The percentage of the other reactions linked to COVID-19 vaccines ranged from 94.2 % to 97.4 %. 

### Spike protein neurotoxicity 

The spike protein notably has a furin cleavage site that allows it to be split into two segments, S1 and S2 (Örd et al., 2020[64]). This is a unique feature of SARS-CoV-2 as compared with its predecessor SARS-CoV. Furin-mediated cleavage of the S1 segment would release it freely into the circulation (Letarov et al., 2021[48]). S1 contains the receptor binding domain (RBD), which binds to the Angiotensin Converting Enzyme 2 (ACE2) receptors on human cells, disabling them (Sui et al., 2021[80]). ACE2 is highly expressed in neurons, and S1 binding to neuronal ACE2 could contribute to neurological symptoms linked to COVID-19 (Xu and Lazartigues, 2022[91]). 

An experiment involving infection of transgenic mice expressing human ACE2 with SARS-CoV-2 demonstrated that the virus was present in the brain, in association with upregulation of the NLRP3 inflammasome. NLRP3 inflammasome activation is a major driver of neurodegeneration (Holbrook et al., 2021[40]). Furthermore, purified SARS-CoV-2 spike glycoprotein primed the inflammasome in microglia through Necrosis Factor-kappa light chain enhancer of activated B cells (NF-κΒ) signaling (Albornoz et al., 2022[6]). 

The S1 subunit of SARS-CoV-2 spike protein, when injected into the hippocampus of mice, induces cognitive deficit and anxiety, along with glial cell activation and neuronal cell death. *In vitro* experiments demonstrated that S1-induced interleukin-1β (IL-1β) release from activated microglia damaged the neurons, causing their death (Oh et al, 2022[63]). When exposed to the spike protein, microglia release pro-inflammatory cytokines which induce an increase in oxidative stress and upregulation of hypoxia inducible factor 1α (HIF-1α) (Clough et al., 2021[25]). HIF-1α is overexpressed in association with many cancers, and it is considered to be an indicator of poor prognosis (Jun et al., 2017[42]). 

### Exosomes, immune suppression, and cancer 

In recent years, researchers are becoming more and more aware of the importance of exosomes in the communication networks among cells (Kalluri and LeBleu, 2020[43]). Exosomes are small extracellular vesicles that are released from nearly all cell types, particularly under stressed conditions (Abels and Breakefield, 2016[2]). It is highly likely that exosomes released by stressed muscle cells in the deltoid muscle transfected with the spike protein mRNA circulate throughout the body, delivering spike protein to distant cells, along with specific microRNAs with regulatory effects. Research has shown that exosomes containing the spike protein can be found in circulation 14 days after mRNA vaccination, and that these exosomes play an important role in the induction of an antibody response (Bansal et al., 2021[15]). The number of exosomes increased after the booster dose and lasted for at least another four months. The inflammatory cytokine TNF-α was significantly elevated in healthy vaccinated subjects compared to controls (p = 0.0078). Exosomes display the spike protein on their surface, and the S1 subunit would be accessible for cleavage by furin and release into the circulation (Bansal et al., 2021[15]).

The exosomes released from transfected cells can contain not only the spike protein but also the complete mRNA sequence encoding it. An important study using mRNA nanoparticles coding for the human protein erythropoietin demonstrated that human cells exposed to lipid nanoparticles containing the mRNA were able to take up the nanoparticles and repackage the mRNA contents into exosomes that were released into the extracellular medium. These exosomes were found to contain a 1:1 molar ratio between mRNA nucleotides and synthetic cationic lipid molecules that had been included in the original nanoparticles. The authors also demonstrated that the exosomes could be taken up by other cells which then produced erythropoietin according to the mRNA code (Maugeri et al., 2019[55]). Trace amounts of the SARS-CoV-2 spike protein mRNA have been found in breast milk (Hanna et al., 2022[38]). These authors wrote: “We speculate that vaccine mRNA released into mammary cell cytosol can be recruited into developing EVs [extracellular vesicles] that are later secreted into EBM [expressed breast milk].” 

Exosomes form the backbone of a communication system among tumor cells, neurons and immune cells in the tumor microenvironment, and the microRNAs they contain can greatly influence the long-term prognosis of the patient (Dragomir et al., 2020[29]). A seminal paper published by Mishra and Banerjea (2021[58]) investigated the potential role of exosomes in the biodistribution of the spike protein produced by the mRNA in the mRNA vaccines. Their *in vitro *studies involved transfecting HEK293T cells with spike mRNA, and then harvesting and analyzing exosomes produced by those cells in response to the transfection. Those exosomes were then presented to microglia grown in culture, and they noted that the exposure resulted in microglial activation and an inflammatory response. These authors wrote: “We propose that SARS-CoV-2 gene product, Spike, is able to modify the host exosomal cargo, which gets transported to distant uninfected tissues and organs and can initiate a catastrophic immune cascade within Central Nervous System (CNS).” 

They further determined that the exosomes contained not only spike protein but also two specific miRNA molecules, miR-148a and miR-590. These two miRNA molecules disrupt the signaling response to type I interferon through suppression of interferon regulatory factor 9 (IRF9), resulting in immune suppression. While HEK293T cells were originally obtained from the kidneys of a fetus, studies have shown that their expression profile is much more typical of neurons than of kidney epithelial cells (Shaw et al., 2002[75]). 

Type I interferons have been shown to inhibit tumor growth by acting on both the tumor and the infiltrating immune cells, and, in fact, there is interest in exploiting type I interferon as a therapeutic agent in cancer therapy (Yu et al., 2022[92]). In a previous paper, we proposed that the mRNA vaccines would accelerate the rate of progression of pre-existing tumors through interference with the type I interferon response mediated by the microRNAs included with the spike protein within exosomes released by transfected cells (Seneff et al., 2022[72]).

### A role for programmed death ligand 1

Programmed Death Ligand 1 (PD-L1) is a trans-membrane protein that is expressed on antigen presenting cells and on cancer cells, and it acts as a mechanism to turn down the gain on an overzealous immune response (Kornepati et al., 2022[46]). When it binds to its receptor PD-1, present on multiple immune cell types, it suppresses their ability to destroy tumor cells and keep cancer in check. Recently, there has been much excitement around the idea of using PD-L1 inhibitors to treat cancer (Blank et al., 2005[17]; Ostrand-Rosenberg et al., 2014[65]; Wu et al., 2019[90]; Alsaab et al., 2017[8]). PD-1 is expressed on activated T cells, natural killer cells, and B cells, as well as lymphocytes, macrophages, dendritic cells and monocytes. PD-1 is upregulated in T cells in the tumor microenvironment, and its interaction with PD-L1 can lead to senescence and impaired antitumor immune responses (Ahmadzadeh et al., 2009[4]). 

A study comparing 37 vaccinated subjects with 15 healthy controls found a statistically significant increase in levels of interferon-γ (IFN-γ) in the subjects, one month after vaccination, compared to the controls (Kurteva et al., 2022[47]). Exposure to IFN-γ induces expression of both PD-L1 and PD-1 in the tumor microenvironment, and this can lead to immune evasion. IFN-γ suppresses miR-513 expression, and miR-513 was the first miRNA that was recognized as a PD-L1 negative regulator, through its direct binding to the 3' UTR of PD-L1 mRNA (Ai et al., 2020[5]). 

A study specifically looking at PD-L1 expression on granulocytes and monocytes taken from 62 vaccinated volunteers compared to 12 controls revealed that the surface expression of PD-L1 was sharply increased just two days post vaccination (Loacker et al., 2022[51]). These authors wrote: “It seems plausible that PD-L1 is upregulated after a strong vaccine-related activation because an activated immune system needs to be regulated to avoid autoimmune collateral damage.” 

In a study involving 40 patients suffering from metastatic basal cell carcinoma, specimens from primary lesions in the tumor microenvironment were examined, and PD-L1 was found to be expressed on tumor cells in 22 % of the samples, and on tumor-infiltrating lymphocytes and associated macrophages in 82 % of the samples (Lipson et al., 2017[50]). This suggests that increased expression of PD-L1 following vaccination may have played a role in the rapid progression of cancer in our patient.

## Discussion

Τhe tumor that produced extensive metastatic disease in our patient was a cutaneous infiltrative basal cell carcinoma that mimicked the aggressiveness of an adnexal neoplasm described recently (Sarangi et al., 2022[70]). But even in these cases of aggressive endocrine mucin-producing sweat gland carcinomas (EMPSGCs), when the primary tumor is surgically excised, the time interval for metastasis is, in the vast majority of cases, prolonged (9-10 years) (Alsaad et al., 2007[9]). However, the severity and metastatic infiltrations to the masseter, parotid gland, zygoma and preauricular area of those cases are overwhelmingly similar to the present case, except for their slow development. Our patient had a significantly elevated D-dimer value, 1523 ng/ml, which is strongly linked to tumor node metastasis, rapid progression of cancer (Dai et al., 2018[27]) and increased mortality during malignancy. In addition, elevated levels of D-dimers are associated with circulating cancer cells during e.g., breast cancer recurrence and have poor prognosis (Ghadhban, 2018[31]; Siddiqui et al., 2021[78]). 

Basal cell carcinomas, only on rare occasions, are also metastatic, particularly in their infiltrative basaloid type forms (Piva de Freitas et al., 2017[66]). Notably, the cutaneous infiltrative basal cell carcinoma in our case was not connecting to epidermal layers, nor was a primary infiltrative tumor identified in the outer layers of the skin, which would have suggested that the patient's tumor was metastatic from a primary carcinoma. This establishes the tumor as an extremely rare entity when compared to other cases of metastatic basal cell carcinomas, especially given the short temporal window within which metastases occurred (Boswell et al., 2006[18]; von Domarus et al., 1984[86]; Seo et al., 2011[73]). Usually, the potential of a basal cell carcinoma is such that it produces secondary metastatic disease after many years of primary tumor (von Domarus et al., 1984[86]; Seo et al., 2011[73]).

Bell's palsy (BP) is widely recognized as a mononeuritic variant of Guillain-Barré syndrome (Greco et al., 2012[33]; Adour et al., 1978[3]). BP involves T-cell-mediated immune responses against the myelin of peripheral cranial VII nerves and autoimmune attack on the nerve. 

After a comprehensive review of the published medical literature, a conclusive answer for the true nature of autoimmune disease in BP remains unanswered. However, it has been confirmed that it involves an immune dysregulation of T cells (Greco et al., 2012[33]; Kim et al., 2021[45]). It is reasonable to think that an immune reactivation of Varicella zoster virus (VZV) infection can trigger autoimmune responses that lead to facial paralysis in some of the BP cases (Abdel-Aziz et al., 2015[1]). However, this was not the case in our patient, as he had not suffered from any herpes virus infection during his malignancy period or in the past, so could not have been experiencing an incidence of VZV reactivation. 

Symptoms attributed to BP had previously been linked to a recurrent invasive basal cell carcinoma arising in an anatomical region that led to development of the characteristic hemilateral facial paralysis (May and Lucente, 1972[56]). BP is a serious immune-mediated adverse event following mRNA vaccination against COVID-19 (Poudel et al., 2022[67]). It is speculated that mRNA vaccines, due to the molecular mimicry of antigens (spike protein of SARS-CoV-2), enhance autoimmune reactivations that lead to BP (Poudel et al., 2022[67]). In this regard, autoimmune mechanisms as shown in the study of Chiaro et al. (2021[23]) and via the influence of molecular mimicry, enhance the anti-cancer immunotherapy against invasive melanoma when PD-1 is blocked. PD-1 as well as PD-2 are regularly expressed on the surface of activated monocytes and antigen-presenting cells (APCs) (including granulocytes) (Alsaab et al., 2017[8]). 

As we stated earlier, it has recently been found that SARS-CoV-2 spike protein mRNA vaccination in humans significantly enhances the expression of PD-L1 on monocytes and granulocytes of vaccinees, just 2 days following mRNA administration. In this regard, an interesting and potentially relevant to our case report concerns a patient diagnosed with Angioimmunoblastic T cell Lymphoma (AITL). He had received two doses of an mRNA vaccination 6 and 5 months earlier. Prior to starting chemotherapy, he received a booster mRNA injection, and within a few days noted progressive swelling of several palpable nodes. A follow-up positron emission tomography/computed tomography (PET/CT) scan was performed 8 days after the booster. Lesions throughout his body were found to be larger and more metabolically active than found in the previous PET/CT performed just 22 days prior. The authors note the potential for the mRNA vaccinations to trigger this unique form of lymphoma, writing, “the supposed enhancing action of the vaccine on AITL neoplastic cells is fully consistent with previous observations identifying Tfh [T follicular helper] cells within germinal centres as key targets of nucleoside-modified mRNA vaccines both in animals and in man.” (Goldman et al., 2021[32]). We proposed that a mechanism underlying the enhanced growth and metabolism of the AITL cells may be due to enhancement of PD-L1 expression on APCs (Loacker et al., 2022[51]). However, further investigations are needed to confirm this hypothesis. The spike protein has clearly been shown in multiple studies to be neurotoxic, and it induces inflammation in the brain, leading to oxidative stress and upregulation of proteins that increase cancer risk.

Overall, the short time frame and extremely invasive characteristics of BCC metastases in our patient suggest that immune system disturbances by the mRNA anti-COVID-19 vaccination may have led to the accelerated progression of the disease. The patient's initial presentation with facial palsy typical of BP served to conceal the underlying malignancy. Had an accurate diagnosis been made sooner, it is possible that an earlier intervention could have prevented this tragic outcome.

## Conclusion

We propose that the simultaneous dysfunction of both the facial and trigeminal nerves, likely through inflammation and T-cell-mediated autoimmunity against myelin of the peripheral nerves, produced an impairment of T cell response and suppressed the innate anti-tumor immune response in our patient, facilitating the basal cell carcinoma metastatic potential. Our group has previously published evidence that the mRNA injections should be expected to suppress innate immunity and thus promote carcinogenesis, primarily through suppression of type I interferon and the multiple downstream repercussions of that suppression (Seneff et al., 2022[72]). Other research has shown the upregulation of PD-L1 in immune cells shortly after mRNA vaccination. This ligand has been shown to suppress the ability of T cells to attack tumors. In closing, it is interesting to note that a therapy that stimulates IFN-α production has shown a high degree of efficacy in the treatment of basal cell carcinoma (Schulze et al., 2005[71]; Singal et al., 2016[79]). The success of this therapy against BCC supports the immune-based model of enhanced malignancy we outline in this paper and points toward additional therapeutic avenues should similar malignancies arise in other patients, subsequent to an mRNA injection.

### Limitations

A serious limitation in the case we report is the refusal of the hospital that performed the biopsy to provide the histopathological images or perform immunohistochemical staining for the spike protein. We provide as supplementary material the histology reports given to us by the patient's relatives and his doctors to enlighten this case further. 

## Declaration

### Conflicts of interest

The authors declare that they have no conflicts of interest.

### Acknowledgment

Stephanie Seneff received funding for this work from Quanta Computers, Inc., Taiwan, under the auspices of the Qmulus project.

## Supplementary Material

Supplementary information

## Figures and Tables

**Table 1:**
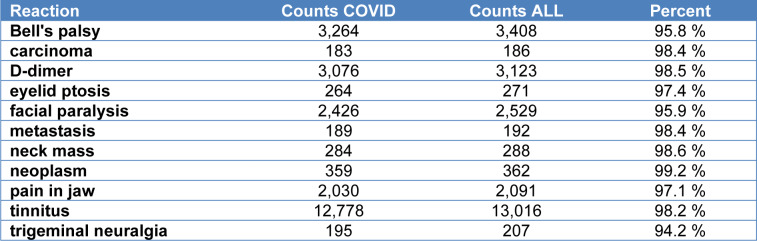
Selected reactions from the VAERS database for the year 2021, comparing counts for US COVID-19 vaccine adverse events with counts for all vaccines during the same time period. These data were derived from an on-line database available at wonder.cdc.gov/vaers.html. Counts COVID: the number of events where COVID vaccine was listed as an administered vaccine in association with the reaction. Counts ALL: The total number of cases for that reaction among ALL the vaccines administered that year.

**Figure 1:**
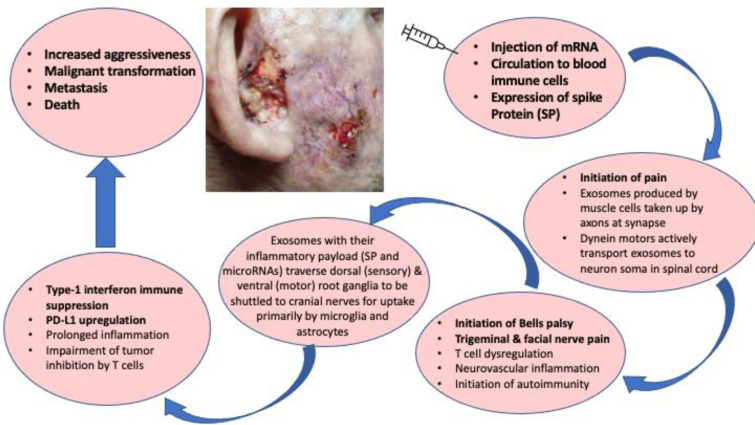
Graphical abstract
