# Supplementary material for: Bell’s palsy or an aggressive infiltrating basaloid carcinoma post-mRNA vaccination for COVID-19? A case report and review of the literature
Source: EXCLI J. 2023 Sep 15;22:992–1011. doi: 10.17179/excli2023-6145 (PMC10620857; doi:10.17179/excli2023-6145)
Supplement: Supplementary information [file EXCLI-22-992-s-001.pdf]

EXCLI Journal 2023;22:992-1011 – ISSN 1611-2156
Received: May 03, 2023, accepted: September 11, 2023, published: September 15, 2023

Supplementary information to:

Case report:

BELL'S PALSY OR AN AGGRESSIVE INFILTRATING BASALOID CARCINOMA POST-MRNA VACCINATION FOR COVID-19? A CASE REPORT AND REVIEW OF THE LITERATURE

Anthony M. Kyriakopoulos[1], Greg Nigh[2], Peter A. McCullough[3], Maria D. Olivier[4], Stephanie Seneff[5*]

[1] Director and Head of Research and Development, Nasco AD Biotechnology Laboratory, Department of Research and Development, Sachtouri 11, 18536, Piraeus, Greece
[2] Naturopathic Oncologist, Immersion Health, Portland, OR 97214, USA
[3] Chief Medical Advisor, Truth for Health Foundation, Tucson, AZ, USA
[4] Director and medical practitioner, Dr. Maré Olivier, Inc., Kuils River, South Africa
[5] Senior Research Scientist, Computer Science and Artificial Intelligence Laboratory, Massachusetts Institute of Technology, Cambridge, MA, USA

* **Corresponding author:** Stephanie Seneff, Computer Science and Artificial Intelligence Laboratory, Massachusetts Institute of Technology, Cambridge, MA, USA;
E-mail: seneff@csail.mit.edu

https://dx.doi.org/10.17179/excli2023-6145

This is an Open Access article distributed under the terms of the Creative Commons Attribution License (http://creativecommons.org/licenses/by/4.0/).

EXCLI Journal 2023;22:992-1011 – ISSN 1611-2156
Received: May 03, 2023, accepted: September 11, 2023, published: September 15, 2023

DEPARTMENT OF ANATOMICAL PATHOLOGY
Tel: 021 938 5226/5350, Fax: 021 938 6559

EPISODE NUMBER: [redacted]

CLINICAL:
A 56 year old male smoker with a suspected right parotid malignancy - 5 month history, severe pain, fixed.
CNV II palsy and Trigeminal neuralgia.
Area of ulceration noted pretragal.
Mass biopsied.

MACROSCOPIC:
The specimen container is topographically unlabelled, but is marked with the patient's details. The specimen consists of multiple small tissue fragments, the largest measuring 3 x 1 x 1mm.
dl/cp

MICROSCOPIC:
Sections show cores of skin tissue fragments exhibiting extensive solar elastosis with associated inflamed granulation tissue. Infiltrating islands composed of basaloid cells with peripheral palisading and stromal clefting are present, but no connection to the overlying epidermis is demonstrated. There is no evidence of parotid parenchyma.

IMMUNOHISTOCHEMICAL STAINS:
p63: positive
CK5: positive
EMA: negative
BER-EP4: positive

COMMENT:
The histomorphologic and immunophenotypic features are most consistent with part of a basal cell carcinoma.

Skin origin from the pretragal ulcerated lesion should be further investigated.

DIAGNOSIS:
Right parotid lesion, biopsy:
- c/w Basal cell carcinoma - please see comment.

Supplementary Figure 1: Immuno-histopathology evaluation of patient biopsy revealed extensive infiltrations of basaloid type cells that formed an invasive basal cell carcinoma

EXCLI Journal 2023;22:992-1011 – ISSN 1611-2156
Received: May 03, 2023, accepted: September 11, 2023, published: September 15, 2023

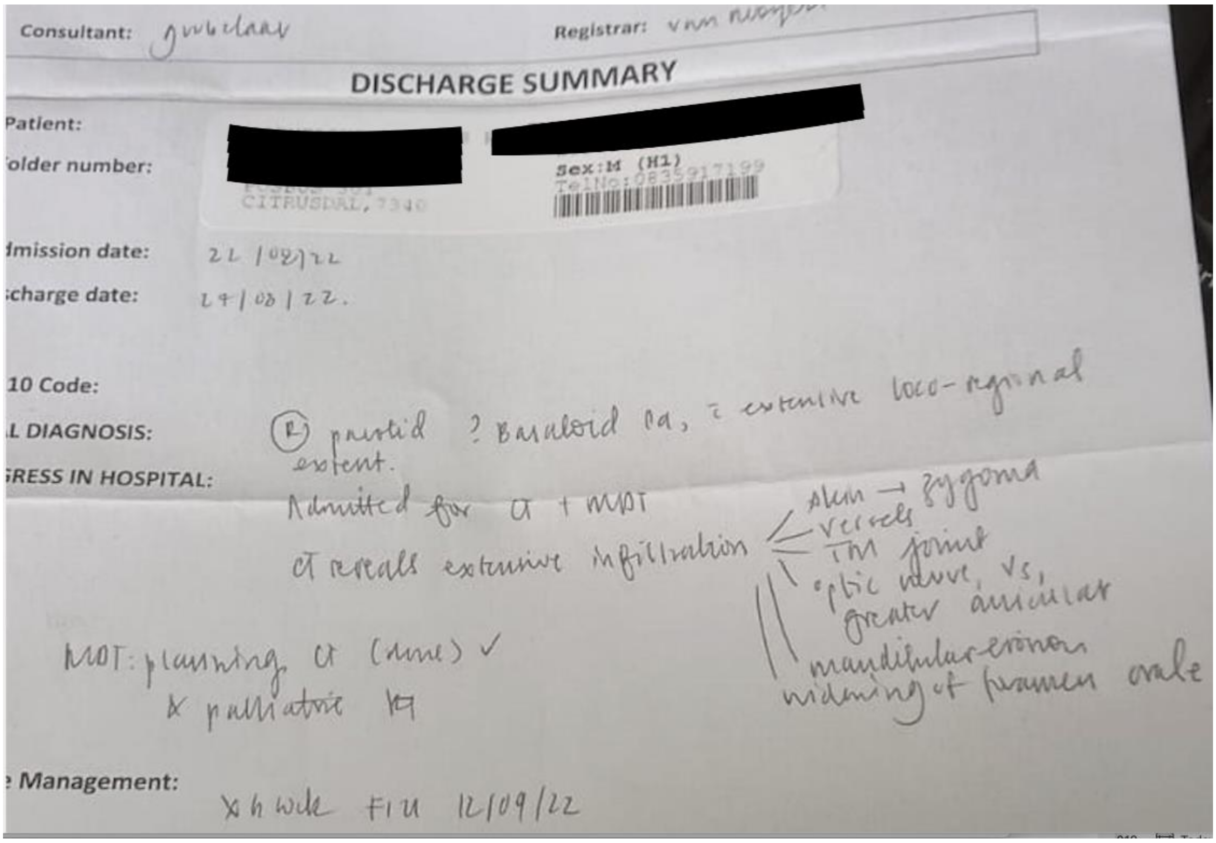

Consultant: [illegible] Registrar: [illegible]

DISCHARGE SUMMARY

Patient: [redacted]
older number:
Sex:M (H1)
TelNo:0835917199
CITRUSDAL, 7340

dmission date: 22/08/22
scharge date: 24/08/22.

10 Code:
L DIAGNOSIS: (R) parotid ? Basaloid Ca, c̄ extensive loco-regional extent.
GRESS IN HOSPITAL: Admitted for CT + MDT
CT reveals extensive infiltration – skin → zygoma, vessels, TM joint, optic nerve, V3, greater auricular, mandibular erosion, widening of foramen ovale

MDT: planning CT (done) ✓
& palliative RT

e Management: 6 week F/U 12/09/22

Supplementary Figure 2: The hospital discharge summary of patient described extensive infiltrations of basaloid type cancer cells inside the zygomatic, temporomandibular joint, optic nerve, fifth cranial nerve and greater auricular nerve. This indicated perineural invasion or spread of cancer to neural tissue. Widening of foramen ovale and mandibular lesions is also described.
